# Using Crocodylians for monitoring mercury in the tropics

**DOI:** 10.1007/s10646-023-02703-1

**Published:** 2023-10-10

**Authors:** Jérémy Lemaire

**Affiliations:** Department of Behavioral and Cognitive Biology, University of Vienna, Djerassiplatz 1, 1030 Vienna, Austria

**Keywords:** Bioindicator, Mercury, Tropical ecosystems, Blood, Keratinized tissues, Goldmining

## Abstract

Mercury contamination is a widespread phenomenon that impacts ecosystems worldwide. Artisanal Small Scale Gold Mining (ASGM) activities are responsible for more than a third of atmospheric Hg emission. Due to Hg toxicity and its broad and elevated prevalence in the environment resulting from ASGM activities in the tropics, its biomonitoring is essential to better understand the availability of its methylmercury (MeHg) form in the environment. The Minamata Convention was ratified with the objective to “*protect human health and the environment from anthropogenic emissions and releases of mercury compounds*”. Biomagnification of MeHg occurs through the trophic food web, where it biomagnifies and bioaccumulates in top predators. To monitor environmental MeHg contamination, studies have evaluated the use of living organisms; however, reptiles are among the least documented vertebrates regarding MeHg exposure. In this review we evaluate the use of crocodylians for Hg biomonitoring in tropical ecosystems. We found that out of the 28 crocodiles species, only 10 have been evaluated regarding Hg contamination. The remaining challenges when using this taxon for Hg biomonitoring are inconsistencies in the applied methodology (e.g., wet versus dry weight, tissues used, quantification method). However, due to their life history traits, crocodylians are particularly relevant for monitoring MeHg contamination in regions where ASGM activities occur. In conclusion and given their ecological and socio-economic importance, crocodylians are at great risk of MeHg contamination and are excellent bioindicators for tropical ecosystems.

## Introduction

Mercury (Hg) is one of the most concerning global contaminants (Chen et al. 2018) and its ecological processes in the Southern Hemisphere are only recently being described (Chen and Evers 2023). Mercury originates from both natural and anthropogenic sources, with artisanal and small-scale gold mining (ASGM) and fossil fuel combustion being the principal sources of its direct release into ecosystems (Mason et al. 1994, 2012; Obrist et al. 2018). Under anoxic conditions, inorganic Hg is methylated via sulfate-reducing microorganisms into methylmercury (MeHg), the most bioavailable and toxic form of Hg (Compeau and Bartha 1985; Benoit et al. 2003; Podar et al. 2015). MeHg bioaccumulates within organisms over time and biomagnifies through the trophic web, where it may reach levels of concern as measured in different tissues of top predators (Lavoie et al. 2013; Eagles-Smith et al. 2018). The methylmercury form is extremely concerning due to its toxicity for human and wildlife. Deleterious effects attributed to MeHg contamination encompass (but are not limited to) immunotoxicity, alteration of neurological capacity and neuro-behavioral function, impairment of reproduction, and offspring quality (Cordier et al. 2002; Basu et al. 2005; Scheuhammer et al. 2007; Tan et al. 2009; Chin et al. 2013; Landler et al. 2017; Maqbool et al. 2017; Morcillo et al. 2017; Evers 2018).

Due to its toxicity and its capacity to bioaccumulate in living organisms, the evaluation of Hg, and in particular MeHg, concentrations in ecosystems is important to understand for regulatory and policy decisions regarding ecosystem and human health (Evers et al. 2016; Gustin et al. 2016). The Minamata convention was ratified with the objectives to “*protect human health and the environment from anthropogenic emissions and releases of mercury compounds*” (United Nations Environment Programme (2013)). To assess its effectiveness, the selection of appropriate bioindicators, such as those that are of high importance as resources for human consumption, and taxa that are at great risk of Hg exposure, are needed (Evers et al. 2016).

In this regard, studies have evaluated the use of living organisms to monitor environmental contamination, with particular attention given to vertebrates (Gómez-Ramírez et al. 2014; Di Marzio et al. 2018; De Paula Gutiérrez, Agudelo (2020); Haskins et al. 2021). The use of bioindicators offers several advantages over standard methods which analyze Hg in soil, sediment, and water. Mercury and MeHg can concentrate in tissues at varying levels, based on the type of tissue. Some including blood, brain, and keratin-based tissues facilitate analytical detection of total Hg as a high percentage of the Hg is in its methyl form (i.e., > 90%). Quantification of total Hg in key tissue types (e.g., those with a high percentage of MeHg) of targeted bioindicator species provides valuable information on MeHg availability in an ecosystem. While fish, birds, and mammals have been extensively used as bioindicators, reptiles have been more infrequently considered in ecotoxicological studies. Despite the increasing number of studies on snakes, turtles and crocodiles showing their capacities to be used as bioindicators species (Burger et al. 2005; Schneider et al. 2011; Lázaro et al. 2015; Lemaire et al. 2018; Haskins et al. 2021), reptilian model species remain underrepresented.

The tropics are particularly affected by Hg contamination, where ASGM represents the major source of emissions (United Nation Environment Programme (2019)). However, studies in tropical regions generally lag behind Northern Hemisphere regions (Carravieri et al. 2013; Albert et al. 2019, Diez et al. 2019, Chen and Evers 2023). Tropical ecosystems contain a high diversity of reptilians which, in regard to their natural history traits, makes them high quality candidates for biomonitoring MeHg contamination. Crocodylians, being top predators inhabiting tropical and sub-tropical ecosystems, have been used in multiple studies and prove to be excellent bioindicators of environmental Hg contamination (Schneider et al. 2015; Nilsen et al. 2017a; Lemaire et al. 2021a). In this review, we will examine the main findings, highlight the remaining challenges, and propose recommendations for using crocodylians to monitor environmental Hg contamination and to contribute to global biomonitoring efforts that will contribute toward the evaluation of the effectiveness of the ‘Minamata Convention on Mercury’.

## Methods

A literature review was conducted on January 30th, 2023, using Scopus, Web of Science, and the Google Scholar database, with the keywords “Crocodylians”, “Mercury”, and “Methylmercury”. To ensure the relevance of the studies, we excluded those that focused solely on mercury contamination in captive crocodiles and instead focused on studies on wild crocodylian populations. While this literature review may not have been exhaustive, it did provide a robust basis for a global comparison of Hg in various tissues of wild crocodylians from around the world.

## Results and Discussion

### Study species and locations

**Fig. 1:**
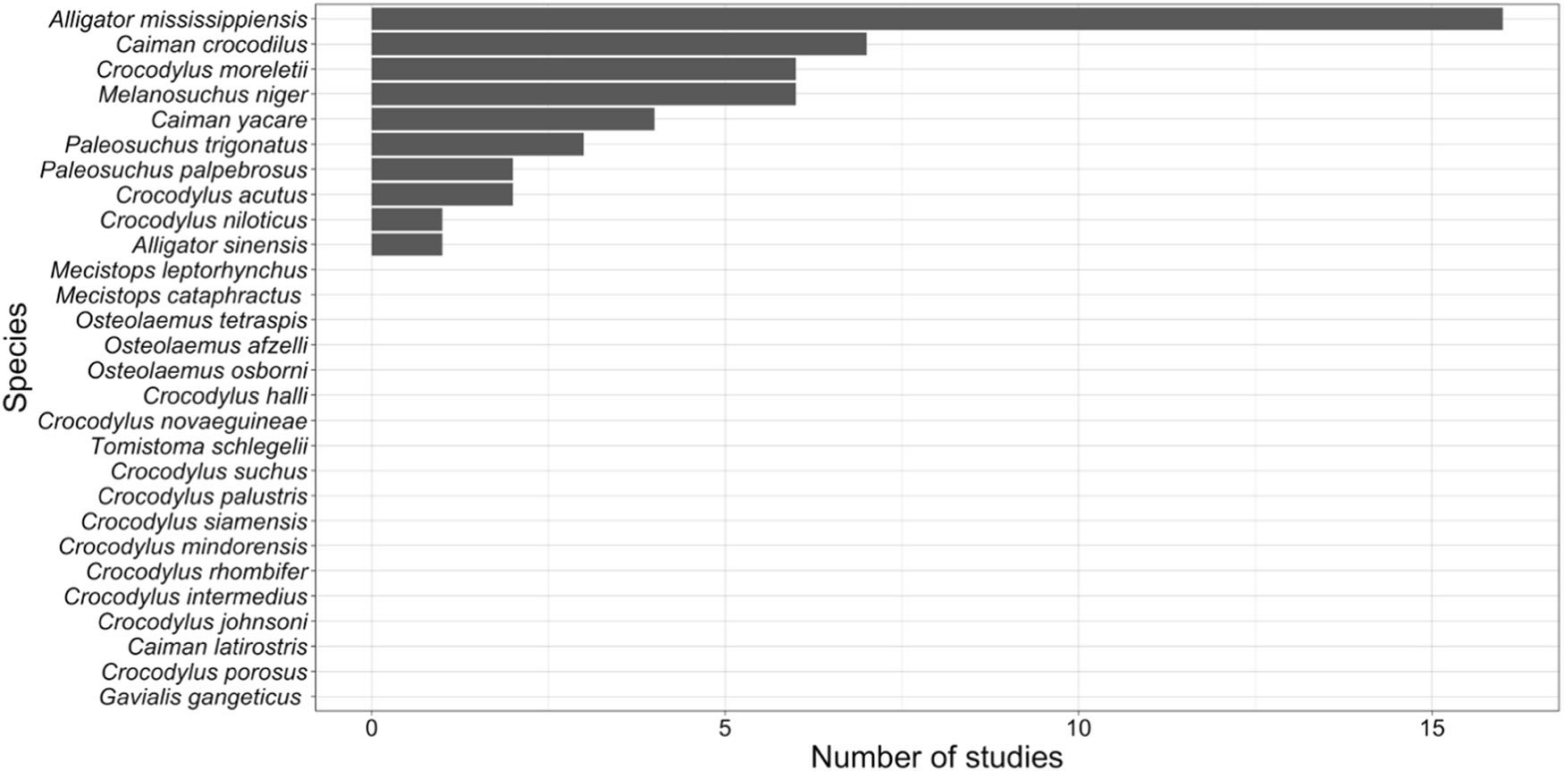
Number of studies on Hg contamination in wild crocodylians which were published by January 30th, 2023

This review demonstrates that although Hg contamination has been documented in crocodylians for approximately 35 years, literature remains limited. In January 2023 only 39 studies reported Hg contamination in wild crocodylid populations, with a recent increase of publications in the last decade. Delany et al. (1988) were the first to publish Hg contamination in muscle tissue of 32 American alligators *Alligator mississippiensis* from various populations in Florida, USA. Since then, out of the 28 extant crocodylid species, Hg contamination has only been documented in 10 of them, accounting for 35,7% (Fig. 1). Among these, three species encompass most of the published studies with *A. mississippiensis* being the most studied species with 16 studies (41%), followed by eight studies (20%) on the Spectacled caiman (*Caiman crocodilus*), and six studies (15%) on the Black caiman (*Melanosuchus niger*) (Fig. 1). In contrast, the Chinese alligator (*Alligator sinensis*) and the Nile crocodile (*Crocodylus niloticus*) have each been the subject of one study (Fig. 1).

**Fig. 2:**
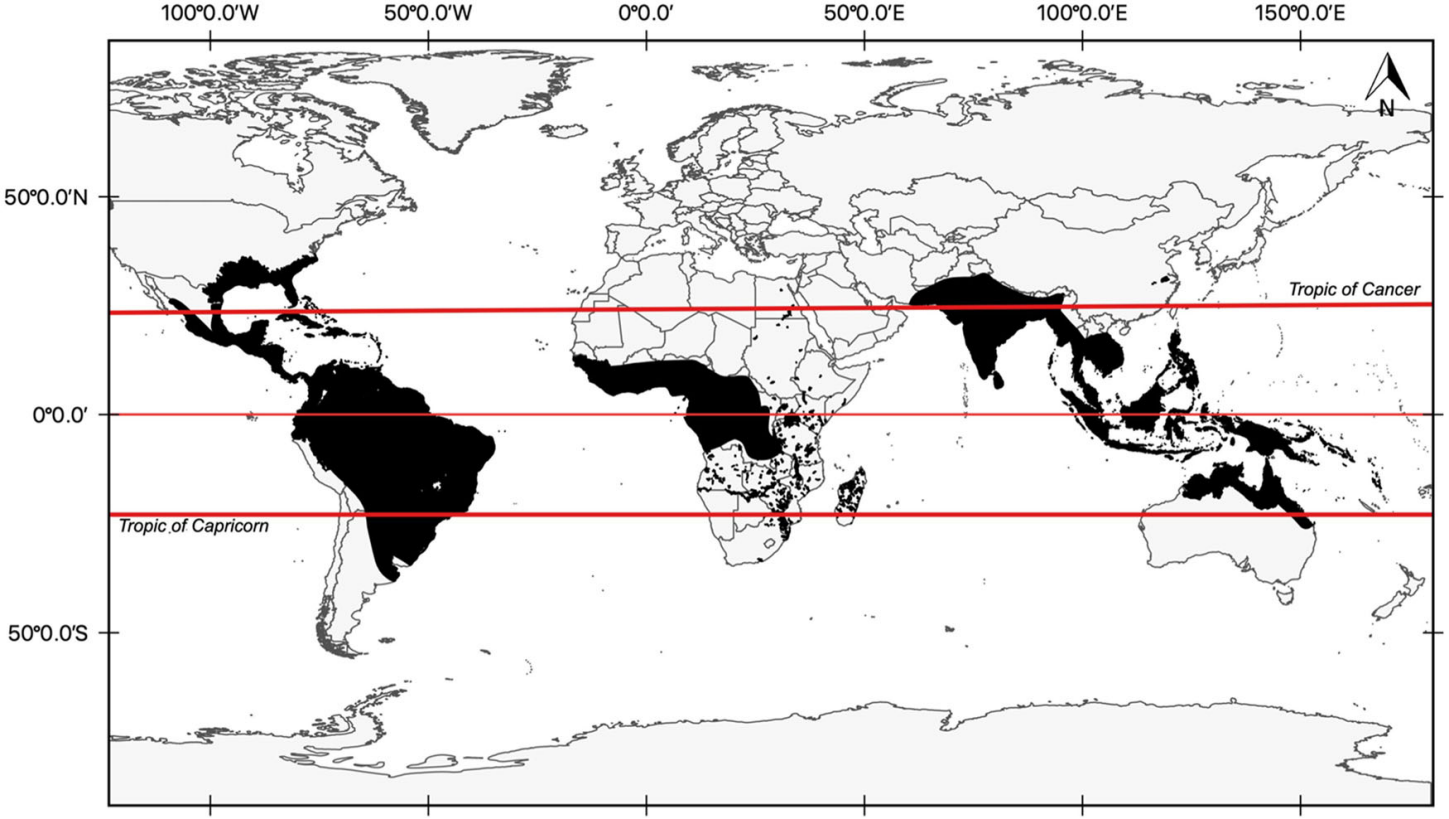
Global distribution range of crocodylian species

Crocodylians are widely distributed across tropical and subtropical ecosystems (Fig. 2). However, most studies on crocodylian Hg contamination have focused on the Americas, while Africa and Asia encompass only two studies where one examined Hg levels in the Chinese alligator (*A. sinensis*) in China, and another in the Nile crocodile (*C. niloticus*) Hg contamination in Zambia (Almli et al. 2005; Xu et al. 2006) (Fig. 2).

Among the investigated species, most of them are species of conservation concern based on the IUCN Red List for Endangered Species assessment, which stresses the need to extend Hg evaluation to all crocodylians to serve as large-scale bioindicators. Additionally, Hg contamination represents an already documented, deleterious impact in archosaurs (Wolfe et al. 1998; Scheuhammer et al. 2007; Ackerman et al. 2016). It has been demonstrated that in alligatorids, Hg contamination, even at low levels, negatively impacts physiological processes such as osmoregulation, hepatic function, and endocrine processes (Lemaire et al. 2021b), damages DNA of erythrocytes (Marrugo-Negrete et al. 2019), alters body condition (Nilsen et al. 2017a), and disrupts embryonic development (Lemaire et al. 2021c). Therefore, it is crucial to gain a better understanding of the threat that Hg contamination poses to crocodylians. The available literature emphasizes the urgent need to better understand its impact on this taxon.

### Mercury quantification

Studies show that in the 10 crocodylian species that have been investigated, Hg concentrations vary according to body size, habitat type, and geographic location. Such findings emphasize the need to better understand how different tissue types correlate with confounding morphometric and environmental factors. Detailed information on Hg concentrations measured in wild populations are summarized in Table 1.

**Table 1:** Total mercury concentration measured in different tissues of crocodylians, Mean ± SD/or SE [Min – Max] in µg.g^−1^, body size Mean ± SD/or SE [Min – Max] in cm, year of collection and location, published between 1988 and January 2023.

Species	Location	Year of collection	*n*	Tissue	Body size	Hg concentration	Authors
American alligator (*Alligator mississippiensis*)	Florida, USA	1985	24	Muscle (ww)	303 cm [290–380]	0.61 [-]	Delany et al. 1988
	Everglades, Florida, USA	1992–1993	12	Liver (ww)	-	39.99 ± 24.05 [8.86–99.48]	Heaton-Jones et al. 1997
	Florida, USA	1992	12	Liver (ww)	-	2.53 ± 4.38 [0.14–16.01]	
	Everglades, Florida, USA	1992–1993	12	Kidneys (ww)	-	25.85 ± 14.63 [5.37–65.53]	
	Florida, USA	1992	12	Kidneys (ww)	-	1.58 ± 2.35 [0.15–9.56]	
	Everglades, Florida, USA	1992–1993	12	Spleen (ww)	-	3.70 ± 3.28 [1.04–13.10]	
	Florida, USA	1992	12	Spleen	-	0.45 ± 0.48 [0.09–1.31]	
	Everglades, Florida, USA	1992–1993	12	Tail muscle (ww)	-	2.61 ± 0.91 [1.11–4.28]	
	Florida, USA	1992	12	Tail muscle (ww)	-	0.33 ± 0.28 [0.04–1.00]	
	Everglades, Florida, USA	1992–1993	12	Leg muscle (ww)	-	2.70 ± 1.41 [0.61–6.05]	
	Florida, USA	1992	12	Leg muscle (ww)	-	0.28 ± 0.19 [0.05–0.60]	
	Everglades, Florida, USA	1992–1993	12	Heart (ww)	-	2.31 ± 1.18 [1.21–4.62]	
	Florida, USA	1992	12	Heart (ww)	-	0.30 ± 0.22 [0.08–0.85]	
	Everglades, Florida, USA	1992–1993	12	Brain (ww)	-	1.37 ± 0.61 [0.52–2.50]	
	Florida, USA	1992	12	Brain (ww)	-	0.16 ± 0.09 [0.03–0.31]	
	Everglades, Florida, USA	1992–1993	12	Spinal cord (ww)	-	1.34 ± 0.57 [0.45–2.55]	
	Florida, USA	1992	12	Spinal cord (ww)	-	0.97 ± 1.64 [0.06–4.98]	
	Everglades, Florida, USA	1992–1993	12	Ovaries (ww)	-	0.70 ± 0.33 [0.39–1.34]	
	Florida, USA	1992	12	Ovaries (ww)	-	1.30 ± 1.64 [0.03–5.91]	
	Everglades, Florida, USA	1992–1993	12	Oviducts (ww)	-	1.19 ± 0.29 [0.89–1.59]	
	Florida, USA	1992	12	Oviducts (ww)	-	1.20 ± 1.87 [0.06–5.42]	
	Everglades, Florida, USA	1992–1993	12	Testes (ww)	-	1.17 ± 0.59 [0.31–2.35]	
	Florida, USA	1992	12	Testes (ww)	-	0.19 ± 0.17 [0.01–0.48]	
	Everglades, Florida, USA	1992–1993	12	Tail scales (ww)	-	1.03 ± 0.42 [0.40–1.86]	
	Florida, USA	1992	12	Tail scales (ww)	-	0.34 ± 0.33 [0.04–1.10]	
	Everglades, Florida, USA	1992–1993	12	Lungs (ww)	-	0.98 ± 0.48 [0.39–1.76]	
	Florida, USA	1992	12	Lungs (ww)	-	0.27 ± 0.20 [0.08–0.63]	
	Everglades, Florida, USA	1992–1993	12	Bile (ww)	-	0.17 ± 0.12 [<0.01–0.53]	
	Florida, USA	1992	12	Bile (ww)	-	0.23 ± 0.20 [0.03–0.59]	
	Everglades, Florida, USA	1994	18	Muscle (dw)	-	5.57 ± 0.47	Jagoe et al. 1998
	Central Florida, USA	1994	21	Muscle (dw)	-	1.85 ± 0.35	
	Okefenokee, South Georgia, USA	1994	9	Muscle (dw)	-	0.80 ± 0.12	
	Savannah River, South Carolina, USA	1994	17	Muscle (dw)	-	4.83 ± 0.88	
	Everglades, Florida, USA	1994	18	Liver (dw)	-	41.03 ± 5.90	
	Central Florida, USA	1994	21	Liver (dw)	-	14.61 ± 3.19	
	Okefenokee, South Georgia, USA	1994	9	Liver (dw)	-	4.30 ± 0.97	
	Savannah River, South Carolina, USA	1994	14	Liver (dw)	-	14.90 ± 2.24	
	Everglades, Florida, USA	1994	17	Kidneys (dw)	-	36.42 ± 53.23	
	Central Florida, USA	1994	21	Kidneys (dw)	-	12.59 ± 2.65	
	Okefenokee, South Georgia, USA	1994	9	Kidneys (dw)	-	4.82 ± 1.34	
	Everglades, Florida, USA	1994	17	Scutes (dw)	-	5.83 ± 1.04	
	Central Florida, USA	1994	20	Scutes (dw)	-	0.52 ± 0.09	
	Okefenokee, South Georgia, USA	1994	9	Scutes (dw)	-	0.29 ± 0.03	
	Savannah River, South Carolina, USA	1994	39	Scutes (dw)	-	5.14 ± 0.64	
	Central Florida, USA	1994	21	Claws (dw)	-	2.69 ± 0.56	
	Okefenokee, South Georgia, USA	1994	9	Claws (dw)	-	1.67 ± 0.16	
	Savannah River, South Carolina, USA	1994	11	Whole blood (dw)	-	2.19 ± 0.38	
	Okefenokee, South Georgia, USA	1994	9	Bone (dw)	-	0.16 ± 0.02	
	Okefenokee, South Georgia, USA	1994	8	Fat (dw)	-	0.19 ± 0.06	
	Okefenokee, South Georgia, USA	1994	9	Spleen (dw)	-	0.63 ± 0.12	
	Okefenokee, South Georgia, USA	1994	9	Brain (dw)	-	0.46 ± 0.14	
	WCA, Everglades, Florida, USA	1994	10	Kidneys (dw)	-	35.00 ± 6.02	Yanochko et al. 1997
	WCA, Everglades, Florida, USA	1994	10	Tail scutes (dw)	-	6.33 ± 1.04	
	WCA, Everglades, Florida, USA	1994	10	Liver (dw)	-	42.15 ± 6.64	
	WCA, Everglades, Florida, USA	1994	10	Muscle (dw)	-	5.68 ± 0.75	
	Holiday Park, Everglades, Florida, USA	1994	7	Kidneys (dw)	-	38.46 ± 9.92	
	Holiday Park, Everglades, Florida, USA	1994	7	Tail scutes (dw)	-	5.12 ± 1.01	
	Holiday Park, Everglades, Florida, USA	1994	8	Liver (dw)	-	39.75 ± 10.01	
	Holiday Park, Everglades, Florida, USA	1994	8	Muscle (dw)	-	5.43 ± 0.53	
	Par Pound, South Carolina, USA	1994	39	Tail scutes (dw)	-	4.58 ± 0.63	
	Par Pound, South Carolina, USA	1994	17	Liver (dw)	-	17.73 ± 2.56	
	Par Pound, South Carolina, USA	1994	21	Muscle (dw)	-	4.08 ± 0.46	
	South Louisiana, USA	1998	42	Muscle (ww)	[124–368]	0.131 [0.047–0.386]	Elsey et al. 1999
	Everglades, Florida, USA	1999	28	Liver (ww)	77.4 ± 9.7 SVL [58.5–93.5]	4.89 ± 3.99 [0.6–17]	Rumbold et al. 2002
	Everglades, Florida, USA	1999	28	Tail muscle (ww)	77.4 ± 9.7 SVL [58.5–93.5]	0.64 ± 0.04 [0.1–1.8]	
	Caddo Lake, Texas / Louisiana, USA	2007	2	Muscle (dw)	149 ± 55.2	0.795 ± 0.010	Chumchal et al. 2011
	Caddo Lake, Texas / Louisiana, USA	2007	2	Liver (dw)	149 ± 55.2	2.263 ± 0.289	
	South Carolina, USA	2008	33	Liver (ww)	234.4 ± 9.2 [152–336]	5.68 ± 1.4	Campbell et al. 2010
	Florida, USA	2009–2010	62	Liver (dw)	[66.5–370]	[0.0522–23.9]	Horai et al. 2014
	Florida, USA	2012	37	Whole blood (ww)	[43.9–153.5] SVL	0.1937 [0.0567–1.380]	Nilsen et al. [Bibr CR72][Bibr CR72]
	Florida, USA	2012	37	Muscle (ww)	[43.9–153.5] SVL	0.2431 [0.0453–1.183]	
	Florida, USA	2012	37	Liver (ww)	[43.9–153.5] SVL	3.5941 [0.5668–14.293]	
	Florida, USA	2012	30	Scutes (ww)	[43.9–153.5] SVL	0.3185 [0.0622–1.9659]	
	Merritt Island, Florida, USA	2007–2014	174	Whole blood (ww)	[87–187.2] SVL	0.152 [0.0358–1.0664]	Nilsen et al. [Bibr CR71][Bibr CR71]
	Yawkey, South Carolina, USA	2011–2014	15	Whole blood (ww)	136 ± 21 SVL [112–183]	0.150 ± 0.049 [0.048–0.238]	Nilsen et al. 2019
	Bear Island, South Carolina, USA	2011–2014	14	Whole blood (ww)	119 ± 22 SVL [80–165]	0.118 ± 0.058 [0.044–0.234]	
	Kissimmee, Florida, USA	2011–2014	12	Whole blood (ww)	129 ± 33 SVL [90–178]	0.393 ± 0.204 [0.185–0.796]	
	Lochloosa, Florida, USA	2011–2014	10	Whole blood (ww)	126 ± 31 SVL [94–180]	0.146 ± 0.067 [0.039–0.251]	
	St. Johns, Florida, USA	2011–2014	11	Whole blood (ww)	136 ± 20 SVL [96–168]	0.153 ± 0.049 [0.079–0.234]	
	Trafford, Florida, USA	2011–2014	12	Whole blood (ww)	121 ± 25 SVL [90–154]	0.194 ± 0.073 [0.067–0.359]	
	Everglades, Florida, USA	2011–2014	14	Whole blood (ww)	110 ± 23 SVL [92–157]	1.364 ± 0.673 [0.438–2.765]	
	Cape Fear River North Carolina, USA	2021	13	Blood (ww)	94.7 ± 37.2 SVL [50.4–190.8]	0.0792 ± 0.0796 [0.0216–0.3355]	Belcher et al. 2022
	Lake Waccamaw, North Carolina, USA	2021	31	Blood (ww)	95.4 ± 27.5 SVL [57.6–148.9]	0.5111 ± 0.2461 [0.1522–0.9459]	
	St. Johns River, Florida, USA	2021	24	Blood (ww)	119.1 ± 31.9 SVL [50.9–163.1]	0.1483 ± 0.0489 [0.0544–0.2443]	
	Florida, USA	-	30	Fat (ww)	-	0.0482 ± 0.0137	Burger et al. 2000
	Florida, USA	-	31	Liver (ww)	-	0.403 ± 0.0801	
	Florida, USA	-	30	Abdominal muscle (ww)	-	0.0756 ± 0.016	
	Florida, USA	-	29	Skin (ww)	-	0.0558 ± 0.0129	
	Florida, USA	-	29	Tail muscle (ww)	-	0.0625 ± 0.0165	
	Florida, USA	-	22	Tail tip (ww)	-	0.0514 ± 0.00869	
	Savannah River, South Carolina, USA	2020–2021	31	Tail muscle (ww)	-	1.31 ± 0.18 [0.077 -4.33]	Kojima et al. 2023
	Savannah River, South Carolina, USA	2020–2021	53	Whole blood (ww)	-	0.938 ± 0.10 [0.076–3.41]	
	Rockefeller Wildlife Refuge, Louisiana, USA	2002	26	Brain (dw)	73.13 ± 4.43 SVL	0.270 ± 0.043 [0.072–1.143]	Moore et al. 2022
	Rockefeller Wildlife Refuge, Louisiana, USA	2002	27	Claws (dw)	73.13 ± 4.43 SVL	0.759 ± 0.130 [0.055–3.789]	
	Rockefeller Wildlife Refuge, Louisiana, USA	2002	27	Front leg muscle (dw)	73.13 ± 4.43 SVL	0.388 ± 0.067 [0.113–1.827]	
	Rockefeller Wildlife Refuge, Louisiana, USA	2002	27	Liver (dw)	73.13 ± 4.43 SVL	3.120 ± 0.760 [0.291–16.87]	
	Rockefeller Wildlife Refuge, Louisiana, USA	2002	27	Gonad (dw)	73.13 ± 4.43 SVL	0.247 ± 0.056 [0.030–1.525]	
	Rockefeller Wildlife Refuge, Louisiana, USA	2002	27	Heart (dw)	73.13 ± 4.43 SVL	0.465 ± 0.088 [0.133–2.458]	
	Rockefeller Wildlife Refuge, Louisiana, USA	2002	27	Jaw muscle (dw)	73.13 ± 4.43 SVL	0.588 ± 0.109 [0.169–3.030]	
	Rockefeller Wildlife Refuge, Louisiana, USA	2002	27	Kidney (dw)	73.13 ± 4.43 SVL	3.183 ± 0.689 [0.488–16.101]	
	Rockefeller Wildlife Refuge, Louisiana, USA	2002	27	Rear leg muscle (dw)	73.13 ± 4.43 SVL	0.425 ± 0.080 [0.128–2.204]	
	Rockefeller Wildlife Refuge, Louisiana, USA	2002	27	Dermal tail scutes (dw)	73.13 ± 4.43 SVL	0.523 ± 0.210 [0.032–5.789]	
	Rockefeller Wildlife Refuge, Louisiana, USA	2002	27	Tail muscle (dw)	73.13 ± 4.43 SVL	0.478 ± 0.090 [0.154–2.455]	
	Rockefeller Wildlife Refuge, Louisiana, USA	2002	27	Blood (ww)	73.13 ± 4.43 SVL	0.122 ± 0.022 [0.029–0.532]	
Chinese Alligator (*Alligator sinensis*)	Changwing Nature Reserve, China	2004	2	Heart (dw)	-	0.347 [0.343–0.350]	Xu et al. 2006
	Changwing Nature Reserve, China	2004	2	Lung (dw)	-	0.327 [0.248–0.405]	
	Changwing Nature Reserve, China	2004	2	Liver (dw)	-	0.559 [0.492–0.626]	
	Changwing Nature Reserve, China	2004	2	Stomach (dw)	-	0.291 [0.232–0.349]	
	Changwing Nature Reserve, China	2004	2	Kidneys (dw)	-	0.902 [0.869–0.935]	
	Changwing Nature Reserve, China	2004	2	Intestine (dw)	-	0.399 [0.389–0.409]	
	Changwing Nature Reserve, China	2004	2	Tracheas (dw)	-	0.120 [0.092–0.147]	
	Changwing Nature Reserve, China	2004	2	Pancreas (dw)	-	0.061 [0.042–0.080]	
	Changwing Nature Reserve, China	2004	2	Reproductive organs (dw)	-	0.059 [0.032–0.085]	
	Changwing Nature Reserve, China	2004	2	Muscle (dw)	-	0.193 [0.105–0.281]	
Spectacled Caiman (*Caiman crocodilus*)	Rio Purus, Brazil	2008	10	Muscle (ww)	75.4 ± 12 SVL [62–98]	0.2912 ± 0.2128 [0.0632–0.6806]	Schneider et al. 2012
	Rio Purus, Brazil	2008	7	Muscle (dw)	75 ± 10 SVL [66–94]	0.234 ± 0.144 [0.132–0.447]	Schneider et al. 2015
	Rio Purus, Brazil	2008	7	Epidermal scale (dw)	75 ± 10 SVL [66–94]	3.350 ± 2.143 [0.500–7.150]	
	Rio Purus, Brazil	2008	7	Bone (dw)	75 ± 10 SVL [66–94]	0.153 ± 0.121 [0.040–0.370]	
	La Mojana, Colombia	2016	45	Blood (ww)	[50–80] TL	0.039 ± 0.030	Marrugo-Negrete et al. 2019
	La Mojana, Colombia	2016	45	Claws (ww)	[50–80] TL	0.647 ± 0.547	
	La Mojana, Colombia	2016	45	Scutes (ww)	[50–80] TL	0.366 ± 0.205	
	La Mojana, Colombia	2016	20	Blood (ww)	[50–80] TL	0.008 ± 0.003	
	La Mojana, Colombia	2016	20	Claws (ww)	[50–80] TL	0.131 ± 0.038	
	La Mojana, Colombia	2016	20	Scutes (ww)	[50–80] TL	0.032 ± 0.006	
	French Guiana	2016–2020	48	Claws (dw)	66.60 ± 24.11 TL [31.0–176.0]	2.692 ± 1.608 [0.321–8.807]	Lemaire et al. [Bibr CR59][Bibr CR54]
	French Guiana	2016–2020	47	Scutes (dw)	66.60 ± 24.11 TL [31.0–176.0]	2.638 ± 1.497 [0.307–7.407]	
	French Guiana	2016–2020	26	Red Blood Cells (dw)	66.60 ± 24.11 TL [31.0–176.0]	0.963 ± 0.612 [0.145–2.244]	
	French Guiana	2016–2020	40	Whole blood (dw)	66.60 ± 24.11 TL [31.0–176.0]	0.605 ± 0.380 [0.089–1.532]	
	French Guiana	2019–2020	21	Whole blood (dw)	35.9 ± 7.7 SVL [20.2–48.5]	0.676 ± 0.414 [0.168–1.532]	Lemaire et al. [Bibr CR54][Bibr CR56]
	Rio Purus, Brazil	-	8	Muscle (ww)	90 ± 14 [62–105]	0.362 ± 0.231 [0.114–0.834]	Eggins et al. 2015
			15	Liver (ww)	90 ± 14 [62–105]	1.701 ± 1.249 [0.035–5.305]	
			11	Blood (ww)	90 ± 14 [62–105]	0.060 ± 0.063 [0.020–0.240]	
			8	Keratin (ww)	90 ± 14 [62–105]	3.527 ± 3.095 [0.576–10.172]	
	French Guiana	-	34	Whole blood (dw)	72.3 ± 24.7 TL [40.6–176.0]	0.61 ± 0.39 [0.09–1.53]	Lemaire et al. 2022
Yacaré (*Caiman yacare*)	La Paz, Bolivia	2007–2008	64	Muscle (ww)	< 180 TL	0.21 ± 0.22	Rivera et al. 2016
	Colorado-Maja lakes, Bolivia	2017	7	Fat (ww)	[177–220]	0.025 ± 0.03	Salazar-Pammo et al. 2021
	Colorado-Maja lakes, Bolivia	2017	7	Muscle (ww)	[177–220]	0.15 ± 0.06	
	Colorado-Maja lakes, Bolivia	2017	7	Kidneys (ww)	[177–220]	0.57 ± 0.30	
	Colorado-Maja lakes, Bolivia	2017	7	Liver (ww)	[177–220]	1.81 ± 0.80	
	Paraguay River, Pantanal, Brazil	-	17	Caudal scutes (ww)	-	0.0957 ± 0.0922	Lázaro et al. 2015
	Bentos Gomes River, Pantanal, Brazil	-	22	Caudal scutes (ww)	-	0.2639 ± 0.1587	
	Paraguay River, Pantanal, Brazil	-	17	Claws (ww)	-	0.8455 ± 0.6227	
	Bentos Gomes River, Pantanal, Brazil	-	22	Claws (ww)	-	1.9447 ± 0.7037	
	Pantanal, Brazil	-	79	Tail muscle (ww)	-	[0.02–0.36]	Vieira et al. 2011
American Crocodile *(Crocodylus acutus)*	Rio Grande Tárcoles, Costa Rica	2003	6	Scutes (ww)	155.7 ± 5.5 SVL [134–172]	0.0935 ± 0.027	Rainwater et al. 2007
	Belize	2019–2019	30	Scutes mix (dw)	-	[0.002–7.33]	Thirion et al. 2022
Morelet’s Crocodile *(Crocodylus moreletii)*	Gold Button Lagoon, Belize	1997–2001	9	Scutes (ww)	89.8 ± 6.7 SVL [65.0–129.5]	0.0987 ± 0.0216	Rainwater et al. 2007
	New River Watershed, Belize	1997–2001	10	Scutes (ww)	104.4 ± 9.6 SVL [59.5–156.7]	0.0727 ± 0.0204	
	Campeche State, Mexico	2012	92	Scutes (dw)	145.6 ± 37.5 SVL [75–288]	5.4 ± 8.3	Trillanes et al. 2014
	Rio Hondo, Mexico	2012–2013	20	Scutes (ww)	[32–190.5]	0.3741 ± 0.4294	Buenfil-Rojas et al. 2015
	Mexico	2016–2018	5	Claws (ww)	-	1.31 ± 0.32	Buenfil-Rojas et al. 2020
	Mexico	2016–2018	50	Scutes (ww)	-	0.27 ± 0.28	
	Mexico	2016–2018	47	Erythrocytes (ww)	-	0.16 ± 0.20	
	Belize	2016–2019	63	Scutes mix (dw)	-	[0.002–1.73]	Thirion et al. 2022
Nile Crocodile *(Crocodylus niloticus)*	Kafue River, Zambia	1998	4	Liver (ww)	[2.7–3.4]	3.5 [0.97–20]	Almli et al. 2005
	Luangwa, Zambia	1998	5	Liver (ww)	[2.0–4.0]	3.7 [2.2–16]	
	Kafue River, Zambia	1998	4	Kidney (ww)	[2.7–3.4]	0.76 [0.60–15]	
	Luangwa, Zambia	1998	5	Kidney (ww)	[2.0– 4.0]	2.7 [1.3–8.7]	
Black caiman *(Melanosuchus niger)*	Rio Purus, Brazil	2008	13	Muscle (dw)	106 ± 28 SVL [87–191]	0.177 ± 0.102 [0.056–0.371]	Schneider et al. 2015
	Rio Purus, Brazil	2008	13	Epidermal scale (dw)	106 ± 28 SVL [87–191]	3.846 ± 2.815 [1.100–10.400]	
	Rio Purus, Brazil	2008	11	Bone (dw)	106 ± 28 SVL [87–191]	0.080 ± 0.093 [0.020–0.380]	
	Rio Purus, Brazil	2008	11	Muscle (ww)	107.5 ± 31.44 SVL [75.3–190.9]	0.1939 ± 0.0962 [0.0694–0.4066]	Schneider et al. 2012
	Kaw-Roura Nature Reserve, French Guiana	2013–2015	72	Whole blood (dw)	143.2 ± 61.3 TL [46.0–326.0]	1.284 ± 0.672 [0.30–3.41]	Lemaire et al. [Bibr CR54][Bibr CR57]
	Mamirauá Reservoir, Brazil	-	60	Muscle (ww)	[107–309]	0.407 ± 0.114 [0.251–0.784]	Correira et al. (2014)
	Rio Purus, Brazil	-	11	Muscle (ww)	102 ± 27 [75–191]	0.176 ± 0.097 [0.057–0.371]	Eggins et al. 2015
	Rio Purus, Brazil	-	11	Liver (ww)	102 ± 27 [75–191]	2.362 ± 2.257 [0.670–7.520]	
	Rio Purus, Brazil	-	12	Blood (ww)	102 ± 27 [75–191]	0.048 ± 0.032 [0.016–0.134]	
	Rio Purus, Brazil	-	13	Keratin (ww)	102 ± 27 [75–191]	2.092 ± 1.052 [0.209–4.029]	
	French Guiana	-	25	Whole blood (dw)	176.4 ± 72.2 TL [71.0–326]	1.56 ± 0.65 [0.54–2.89]	Lemaire et al. 2022
Dwarf Caiman *(Paleosuchus palpebrosus)*	French Guiana	2016–2020	13	Claws (dw)	79.42 ± 33.22 TL [34.2–150]	8.351 ± 4.965 [2.028–20.042]	Lemaire et al. [Bibr CR54][Bibr CR54]
	French Guiana	2016–2020	13	Scutes (dw)	79.42 ± 33.22 TL [34.2–150]	7.647 ± 4.742 [0.789–15.628]	
	French Guiana	2016–2020	6	Red Blood cells (dw)	79.42 ± 33.22 TL [34.2–150]	2.364 ± 1.884 [0.447–5.775]	
	French Guiana	2016–2020	7	Whole blood (dw)	79.42 ± 33.22 TL [34.2–150]	1.376 ± 0.986 [0.540–3.415]	
	French Guiana	-	5	Whole blood (dw)	75.3 ± 44.6 TL [35.5–150]	1.50 ± 1.18 [0.147–7.509]	Lemaire et al. 2022
Smooth-fronted caiman (*Paleosuchus trigonatus*)	French Guiana	2016–2020	50	Claws (dw)	62.11 ± 36.42 TL [22.8–143]	2.420 ± 1.905 [0.147–7.509]	Lemaire et al. [Bibr CR56][Bibr CR54]
	French Guiana	2016–2020	48	Scutes (dw)	62.11 ± 36.42 TL [22.8–143]	3.332 ± 3.066 [0.087–9.859]	
	French Guiana	2016–2020	11	Red Blood Cells (dw)	62.11 ± 36.42 TL [22.8–143]	0.447 ± 0.270 [0.049–0.774]	
	French Guiana	2016–2020	24	Whole blood (dw)	62.11 ± 36.42 TL [22.8–143]	0.300 ± 0.178 [0.032–0.738]	
	French Guiana	2017–2020	38	Claws (dw)	[23.0–26.5] TL	[0.171–0.663]	Lemaire et al. [Bibr CR54][Bibr CR59]
	French Guiana	2017–2020	38	Scutes (dw)	[23.0–26.5] TL	[0.092–0.251]	
	French Guiana	-	20	Whole blood (dw)	82.8 ± 32.7 TL [27–143]	0.35 ± 0.15 [0.10–0.70]	Lemaire et al. 2022

*ww* wet weight, *dw* dry weight.

A variety of tissues have been analyzed to investigate Hg contamination in crocodylians including blood, muscle, internal organs, and keratinized tissues (e.g., scutes). However, due to the limited literature available and the diversity of matrices studied, straightforward comparisons among studies and species is currently challenging. Moreover, the methodology for sample preparation varies among studies. A major challenge that hinders robust comparison across species, tissues, and locations is the methodology of Hg quantification, which can be reported in wet or dry tissue weight. Several studies have demonstrated that moisture content in crocodylian tissues varies greatly among species and locations, and can affect the interpretation of findings. Therefore, dry weight analysis appears to be essential for inter- and intraspecific comparison (Yanochko et al. 1997; Jagoe et al. 1998; Lemaire et al. 2021d).

Crocodylians are often a source of bushmeat for local communities and the consumption of their meat has been found to pose a health risk due to high Hg levels. Studies have focused on muscle tissue of crocodylians, which showed Hg concentrations as high as 4.28 µg.g^−1^ (ww) in muscles of *A. mississippiensis* (Heaton-Jones et al. 1997), exceeding the WHO recommendation limit of 0.5 µg.g^−1^ for safe consumption (WHO, World Health Organization (2011)).

Monitoring programs are needed to assess the risks associated with crocodylian meat consumption (Elsey et al. 1999; Vieira et al. 2011; Kojima et al. 2023), especially since muscle tissue contains over 70% of the methyl form (Vieira et al. 2011). However, the risk of Hg poisoning is strongly linked to intake frequency (Chételat et al. 2020). The liver plays a crucial role in detoxifying Hg and as a result, MeHg represents less than 40% of total Hg (THg) in liver tissues (Vieira et al. 2011). Analysis of total Hg is a cost-effective way of assessing MeHg levels in muscle tissue. While the MeHg concentration ratio in blood and keratinized tissues has been studied in other species, it has not been measured in crocodylians. Nevertheless, blood and keratinized tissues can serve as a proxy for concentrations of the methyl form with over 80% of total Hg found as MeHg (Oliveira Ribeiro et al. 1999; Renedo et al. 2017; Chételat et al. 2020). This approach can be useful in terms of analytical efficiency (time and costs).

Crocodylian tissues Hg concentrations are not confounded by sex of the individual (e.g., Elsey et al. 1999; Vieira et al. 2011; Lemaire et al. 2021a). While maternal transfer has been shown in some crocodylian species such as *A. mississippiensis* (Nilsen et al. 2020) and *P. trigonatus* (Lemaire et al. 2021c), this phenomenon does not seem to affect Hg concentration in the blood of the females. Therefore, the entire population can be used for monitoring, regardless of sex, which is often difficult to determine in the field. This further facilitates harmonized comparison between studies.

To minimize impact on individuals, less-invasive sampling methods are preferred. Blood, and keratin tissues such as claws and scutes, are obtained via non-lethal sampling methods and provide information on the availability of MeHg in the environment. These tissues are known to be good predictors of Hg concentrations for internal tissues, such as liver and muscle (Jagoe et al. 1998; Burger et al. 2000). This is particularly important given the conservation status of most crocodylid species.

Clipping tail scutes is a commonly used sampling methods for crocodiles, as it further serves for individual identification and can be used for stable isotopes, DNA, and contaminant analysis (De Thoisy et al. 2006; Rainwater et al. 2007; Machkour-M’Rabet et al. 2009; Radloff et al. 2012; Trillanes et al. 2014; Pacheco-Sierra et al. 2016; Santos et al. 2018). Scutes and claws are keratin-rich excretion tissues that display high Hg concentrations due to strong affinity of Hg to sulfhydryl-groups contained in keratin (Alibardi 2003; Alibardi and Toni 2007). Mercury levels in keratinized tissues are considered to reflect long term contamination of the individual (Lázaro et al. 2015; Schneider et al. 2015; Marrugo-Negrete et al. 2019; Lemaire et al. 2021d). However, throughout the multiple studies which have been using scutes, only the studies from Schneider et al. (2015) and Lemaire et al. (2021d) give detailed information on the actual part of the scute that had been used for analysis. The importance of specification of the analyzed tissues was demonstrated in the study from Lemaire et al. (2021d): The authors compared Hg concentration when the total scute was analyzed to only the keratin layer of the scute, and show that bone inclusion lead to an underrepresentation of Hg quantification (in the evaluation of the actual environmental contamination status). This highlights the importance of using only the keratin layer for analysis as proxy of long-term contamination.

In contrast, blood is another valuable tissue for biomonitoring studies, as it is involved in MeHg transportation to organs and reflects recent MeHg uptake directly related to the individual’s diet (Lemaire et al. 2022; Manrico et al. 2017). In this order, ontogenetic dietary shift between juveniles and adults greatly influences measured total Hg concentration in blood, leading to a high variability in the relation between size and MeHg concentration. This ontogenetic shift explains why the relationship between blood Hg and body size is variable and was found in some studies (Eggins et al. 2015; Buenfil-Rojas et al. 2018; Lemaire et al. 2021a), but not in others (Yanochko et al. 1997; Eggins et al. 2015; Lawson et al. 2020). Therefore, when comparing Hg concentrations between different geographic areas, it is important to consider size of individuals. This emphasizes that keratinized tissues and blood are complementary in Hg biomonitoring studies as they reflect long-term and short-term contamination, respectively.

### Pattern of Hg concentrations

Among the available literature, scutes have been the most studied tissue in regard to species diversity. Eight crocodylian species from North and South America have been investigated, with mean Hg concentrations in scutes ranging from 0.188 to 7.647 µg.g^−1^ dw (Table 2).

**Table 2:** Mean of total mercury concentrations ± Standard Deviation measured in scutes of crocodylians (µg.g^−1^ dry weight), compiled from the available literature, per species.

Species	Mean Hg concentration (µg.g^−1^)	References
**Dwarf caiman** (*Paleosuchus palpebrosus*)	7.647 ± 4.742	Lemaire et al. [Bibr CR54][Bibr CR54]
**American alligator** (*Alligator mississippiensis*)	4.069 ± 2.973	Heaton-Jones et al. 1997; Yanochko et al. 1997; Jagoe et al. 1998; Burger et al. 2000; Nilsen et al. [Bibr CR72][Bibr CR72]; Moore et al. 2022
**Black caiman** (*Melanosuchus niger*)	3.701 ± 0.205	Schneider et al. 2015; Eggins et al. 2015
**Smooth-fronted caiman** (*Paleosuchus trigonatus*)	3.332 ± 3.066	Lemaire et al. [Bibr CR57][Bibr CR54]
**Spectacled caiman** (*Caiman crocodilus*)	1.661 ± 1.582	Schneider et al. 2015; Eggins et al. 2015; Marrugo-Negrete et al. 2019; Lemaire et al. [Bibr CR56][Bibr CR54]
**Morelet’s crocodile** (*Crocodylus moreletii*)	1.407 ± 2.246	Rainwater et al. 2007; Trillanes et al. 2014; Buenfil-Rojas et al. 2015; Buenfil-Rojas et al. 2020
**Yacaré** (*Caiman yacare*)	0.297 ± 0.196	Lázaro et al. 2015
**American crocodiles** (*Crocodylus acutus*)	0.188 ± 0.005	Rainwater et al. 2007

Conversion rate from original data in wet weight to dry weight were calculated from the moisture content available for scutes for each species (Yanochko et al. 1997; Lemaire et al. 2021d)

*P. palpebrosus* showed the highest Hg concentrations in scutes, followed by *A. mississippiensis*, *M. niger* and *P. trigonatus* (all > 3.3 µg.g^−1^ dw, Table 2). In contrast, *C. acutus* showed the lowest Hg concentration with 0.188 µg.g^−1^ dw.

The difference in Hg concentrations between species can be explained by their trophic ecology. As for other taxa, one of the sources of variation in Hg concentration in crocodylians is related to their trophic ecology (e.g., trophic position and foraging habitat, Lemaire et al. 2022). Additionally, geographic location plays a predominant role in Hg concentrations, depending on geological background and pollutant activities (Siqueira et al. 2018).

Among the four most contaminated crocodylian species, three are from the Amazon, a region known to present high geological Hg background, often enhanced by mining activities (United Nation Environment Programme (2019); Crespo-Lopez et al. 2023), factors known to increase Hg in trophic food webs. Crocodylian species from the Guiana Shield have the highest reported mean Hg concentrations so far, which is not surprising regarding the Hg-rich geological background and gold mining activities in the region (Rahm et al. 2015; Kroonenberg et al. 2022).

*A. mississippiensis* is the species with the second highest Hg concentration, which can be explained by the habitats where data were collected: *A. mississippiensis* lives in North America and is often encountered in close vicinity of anthropized areas (Beal and Rosenblatt 2020). Further, the species is found in the Florida Everglades, an ecosystem known to have high Hg concentrations (Janssen et al. 2022).

Some crocodylian species seem more likely to be contaminated with Hg depending on their geographical range and their trophic ecology, in this order, it would be of great importance to assess Hg geological background, trophic position and foraging ecology when monitoring Hg contamination to better understand the pattern of Hg concentrations in crocodylians.

## Crocodylians to monitor mercury contamination in the tropics

Due to their unique life history traits, crocodylians (alligators, caimans, true crocodiles, and gharials) are excellent indicators of MeHg contamination. They are high trophic-level predators living in aquatic ecosystems (Somaweera et al. 2020) which favors MeHg contamination due to biomagnification; they are long-lived animals (up to several decades) with a low metabolic, and a high tissue conversion rate (Garnett 1986; Webb et al. 1991) which favors MeHg bioaccumulation; they are territorial and sedentary (e.g., Hutton 1989; Fujisaki et al. 2014; Caut et al. 2019), which allows for precise spatial biomonitoring of MeHg contamination. Due to their large body size and robustness, sample collection is relatively straightforward. Additionally, the distribution of crocodylians across tropical and subtropical ecosystems is vast (Martin 2008), making them excellent candidates for large-scale monitoring. Overall, crocodiles represent a valuable tool in assessing the extent of MeHg availability in aquatic ecosystems.

Artisanal small-scale gold mining (ASGM) activities are among the main sources of Hg contributions to ecosystems, particularly in tropical and subtropical regions such as South America, Africa, and South-East Asia. The estimated annual release of Hg from ASGM activities exceeds 2000 tons each year (United Nation Environment Programme (2019)), with ASGM being responsible for up to 80% of local Hg emissions in Sub-Saharan Africa and 70% in South America (United Nation Environment Programme (2019)). Together, these two regions represent 70% of total Hg emission related to ASGM worldwide (United Nation Environment Programme (2019)). Unfortunately, there is a lack of biotic Hg data for many countries where ASGM activities are widely used (Kom et al. 1998; Peplow and Augustine 2014; Markham and Sangermano 2018, United Nation Environment Programme (2019)). Given the geographic range of ASGM activities, finding a suitable bioindicator to monitor Hg contamination is challenging. Crocodylians are an ideal candidate for several reasons, including a manageable number of species, their life history traits, and the potential to represent a broad contamination gradient.

Monitoring Hg contamination in crocodylians can help to assess the effectiveness of global biomonitoring. While reptiles are not listed as focal taxa within the Minamata Convention (Evers et al. 2016), there is still tremendous value in understanding MeHg availability to crocodylians and the potential adverse impacts on their behavior, physiology and reproductive success. Sample collection from crocodylians is (relatively) straightforward as there are sufficient tissues (e.g., blood and scutes) that can be sampled non-lethally to monitor Hg contamination over different time scales (see 'Methods'), including retrospective Hg analysis via museum specimens. Using crocodylians as bioindicators can help us to understand the extent and impact of Hg contamination from ASGM activities, and to work towards understanding its harmful effects on both wildlife and human populations, even more in areas where geological Hg is naturally high (e.g., Guyana Shield).

## Recommendations

The present review highlights several biases that limit comparisons among studies, species, and tissues in the assessment of Hg contamination in crocodylians. One of these biases is the quantification of Hg concentrations in wet- or dry weight, which can be tissue-specific and the variability among individuals and location as shown in *A. mississippiensis* (Yanochko et al. 1997; Jagoe et al. 1998), *P. palpebrosus*, *C. crocodilus*, and *P. trigonatus* (Lemaire et al. 2021d). To enable rigorous comparison of Hg contamination among species and locations, the use of dry samples should be emphasized.

The use of keratinized tissues such as scutes provides valuable information on long-term Hg contamination of an individual via a minimally invasive method. While this tissue has been used in many studies, the actual part of the scute used during analytical procedure is not yet standardized. Scutes are used as keratinized tissues to study the long-term accumulation of Hg. However, as first highlighted by Schneider et al. (2015) and then demonstrated by Lemaire et al. (2021d), using full scutes results in variation of measured Hg concentrations due to the integration of unknown quantities of bone and connective tissues. To avoid this bias, only the keratin layer should be used when scutes are used to quantify Hg contamination.

This review also reveals that in 18 crocodylid species, Hg contamination has never been assessed. Filling this knowledge gap should be a priority, particularly for species which are strongly impacted by human activities (e.g., mining, oil extraction, and urbanization), which can increase the bioavailability of MeHg in the environment. Furthermore, ecotoxicological studies on potential physiological, behavioral, and reproductive effects of MeHg contamination are needed.

In conclusion, and given their ecological and socio-economic importance, crocodylians are at great risk of Hg contamination, and are excellent bioindicators to global biomonitoring interest for MeHg by the Minamata Convention on Mercury. To ensure robust and standardized assessments of Hg contamination, future studies should consider the methodological points in this review.
